# Radical scavenging activity of *Chlorophytum borivilianum* L. root extract and its protective role in cauda epididymal sperm integrity in *Mus musculus* after gamma irradiation

**DOI:** 10.3389/fcell.2023.1064574

**Published:** 2023-03-21

**Authors:** Ruchi Vyas, Kavindra Kumar Kesari, Norbert Lukac, Petr Slama, Shubhadeep Roychoudhury, Rashmi Sisodia

**Affiliations:** ^1^ Department of Zoology, University of Rajasthan, Jaipur, India; ^2^ Department of Applied Physics, School of Science, Aalto University, Espoo, Finland; ^3^ Faculty of Biotechnology and Food Sciences, Slovak University of Agriculture in Nitra, Nitra, Slovakia; ^4^ Laboratory of Animal Immunology and Biotechnology, Department of Animal Morphology, Physiology and Genetics, Faculty of AgriSciences, Mendel University in Brno, Brno, Czech Republic; ^5^ Department of Life Science and Bioinformatics, Assam University, Silchar, India

**Keywords:** sperm maturation, sperm characteristics, oxidative stress, *Chlorophytum borivilianum*, male fertility

## Abstract

**Background:** *Chlorophytum* *borivilianum* L. is a recognized herbal medicine for the management of impotency in South Asian countries. In *Ayurveda*, it is used for the management of multiple health conditions, including diabetes, infection, and cardiovascular diseases. Parts of the plant have been used as excellent antioxidants and scavengers of free radicals. Since oxidative stress plays an important role in spermatogenesis and fertility in male populations, this study evaluated the role of ethanolic extract of *C. borivilianum* roots in epididymal sperm maturation against adversities posed by ionizing gamma irradiation.

**Materials and methods:** Antioxidant potential of *C*. *borivilianum* root extract (CRE) was evaluated through DPPH (2,2-diphenylpicrylhydrazyl) and NO (nitric oxide) scavenging assays. Four groups of healthy Swiss albino mice were constituted, which were labeled as follows: Group I: sham control, Group II: 7-day pre-treatment with 50 mg/kg CRE, Group III: 6 Gy irradiation without pre-treatment, and Group IV: 7-day pre-treatment with 50 mg/kg CRE and 6 Gy irradiation on day 7. Swiss albino mice were observed for 30 days and later sacrificed to evaluate sperm quality parameters.

**Results:** CRE showed a remarkable antioxidant potential with IC_50_ values of 46.37 μg/ml and 98.39 μg/ml for DPPH and NO, respectively. A significant decline (*p* < 0.001) in cauda epididymal sperm count, motility, and viability was observed in Group III animals. Group IV also showed a substantial decline (*p* < 0.01) in all three parameters compared to Group I; nonetheless, these were significantly higher than Group III. Morphological alterations indicated a coiled and bent tail, with the presence of cytoplasmic droplets in Group III, which declined substantially in Group IV. The ultrastructure of sperm indicated higher curvature of hook in Group III than Group IV, indicating specific interferences in the sperm maturation process.

**Conclusion:** It was concluded that pre-treatment with 50 mg/kg body weight of CRE could protect sperm during epididymal maturation against oxidative stress.

## 1 Introduction

*Chlorophytum borivilianum* L., commonly known as *Shweta muesli*, is a medicinal plant found abundantly in the Indian subcontinent. The all-natural traditional medicinal system called *Ayurveda* was developed in India more than 3,000 years ago. The Sanskrit terms *ayur* (life) and *veda* (knowledge) are used to form the name *Ayurveda* (the science or knowledge of life). Interestingly, in *Ayurveda*, *C*. *borivilianum* L. is considered an important medicine in the *Vajikarana* (sexual enhancement and improved progeny) segment (Agnivesha and Dridhabala, 2006). Several studies reported that *C. borivilianum* plays an important role in rejuvenation (Miraj et al., 2020; Verma et al., 2020) and revitalization characteristics to improve sexual dynamics (Puri, 2003) and alleviates sexual dysfunction. Furthermore, various parts of *C*. *borivilianum* are prescribed by *Ayurveda* practitioners for improving health issues including diabetes, infections, and cirrhosis, among others (Singh et al., 2011). Regardless, it is mostly used to treat impotency and infertility. Kenjele et al. (2008) reported that dried roots of *C*. *borivilianum* L. can effectively enhance sexual arousal, vigor, and libido in rats. Giribabu et al*.* (2014) reported that roots of *C*. *borivilianum* can prevent the decrease in sperm count, motility, and viability induced by streptozotocin. A previous study indicated that *C*. *borivilianum* root extract (CRE) can better equip testicular cells against intense oxidative stress (Vyas et al., 2022). *C*. *borivilianum* usually contains stigmasterol, neotigogenin, hecogenin, alkaloids, phenolic acid, flavonoids, saponins, and palmitic acid (Visavadiya et al., 2010; Giri et al., 2015). Among these, stigmasterol (Pratiwi et al., 2021), alkaloids (Macáková et al., 2019), flavonoids (Jovanovic et al., 1996), and saponins (Khan et al., 2022) have been strongly associated with the reduction of oxidative stress and inhibition of ROS generation. Recently, in our previous study, nine compounds have been identified, including 5-methylhex-2-yl pentadecyl ester (23.69%), 9,10-anthracenedione (19.02%), and phthalic acid (18.63%) (Vyas et al., 2020). These compounds are known to be primarily responsible for antioxidant potential (Govindarajan et al., 2005) and alleviation of oxidative stress (Devi et al., 2021).

Sperm oxidative damage is one of the underlying mechanisms of male infertility (Aitken et al., 2014). Polyunsaturated fatty acids lead to the deposition of linoleic acid, facilitating lipid peroxidation (LPO) (Brash, 1999; Maccarrone et al., 2001). The plasma membrane of sperm contains high polyunsaturated fatty acids, thus making it vulnerable to oxidative damage. Before ejaculation, sperm stay transitorily in the cauda epididymis; during this period, an oxidative insult can reduce fertility by many folds. Wu et al. (2020) noted that oxidative stress in cauda epididymal sperm can significantly reduce motility and induce high oxidation of DNA.

Ionizing radiation is widely used in medical diagnosis and as a disinfectant and detector. Nevertheless, accidental exposure or intentional use of ionizing radiation causes immense oxidative stress due to radiolysis of water molecules (Datta et al., 2012). Radiation generates a rapid and large number of reactive oxygen species (ROS), which includes hydroxyl radicals, ionized water, hydrogen radicals, hydrated electrons, superoxide radicals, and hydrogen peroxide (Singh and Singh, 1983). Therefore, ionizing radiation can be used to examine the ameliorating effect of *C*. *borivilianum*, as claimed by previous reports (Vyas et al., 2020 & Vyas et al., 2022). In the present study, the ROS scavenging property of CRE was examined. The results of antioxidant activities of CRE were further associated with sperm quality parameters and amelioration of ionizing radiation-induced damage in cauda epididymal sperm.

## 2 Materials and methods

### 2.1 Test material

Commercially available CRE from Naturemed, Hyderabad, India, was used for this study. The extract was verified by the Department of Botany, University of Rajasthan, Jaipur, India (RUBL No. 19902). The working concentration of the extract was prepared by adding 5% CRE in 250 ml of distilled water. This solution was allowed to boil for 1 h at 70°C. Whatman No. 1 filter paper was used to remove impurities. Later, the clear solution was stored at 4°C in the refrigerator until further use.

### 2.2 Animal model and ethical approval

Swiss albino mice (*Mus musculus*), aged 6–8 weeks and weighing 20–30 g, were used. The animals were housed in a departmental facility under constant observation of veterinary experts. Randomly bred mice were maintained in equal ratios of light and dark periods. The mice were fed with a pellet diet, and water was provided *ad libitum*. The study was approved by the Institutional Animals Ethics Committee (IAEC), and the procedures were followed under the strict guidance of the Indian National Science Academy (INSA) and the Committee for Purpose of Control and Supervision of Experiments and Animals (CPCSEA, 2010) (IAEC Approval No.: 1678/90/re/S/12/CPCSEA, dated 16 June 2017).

### 2.3 Experimental design

**FIGURE 1:**
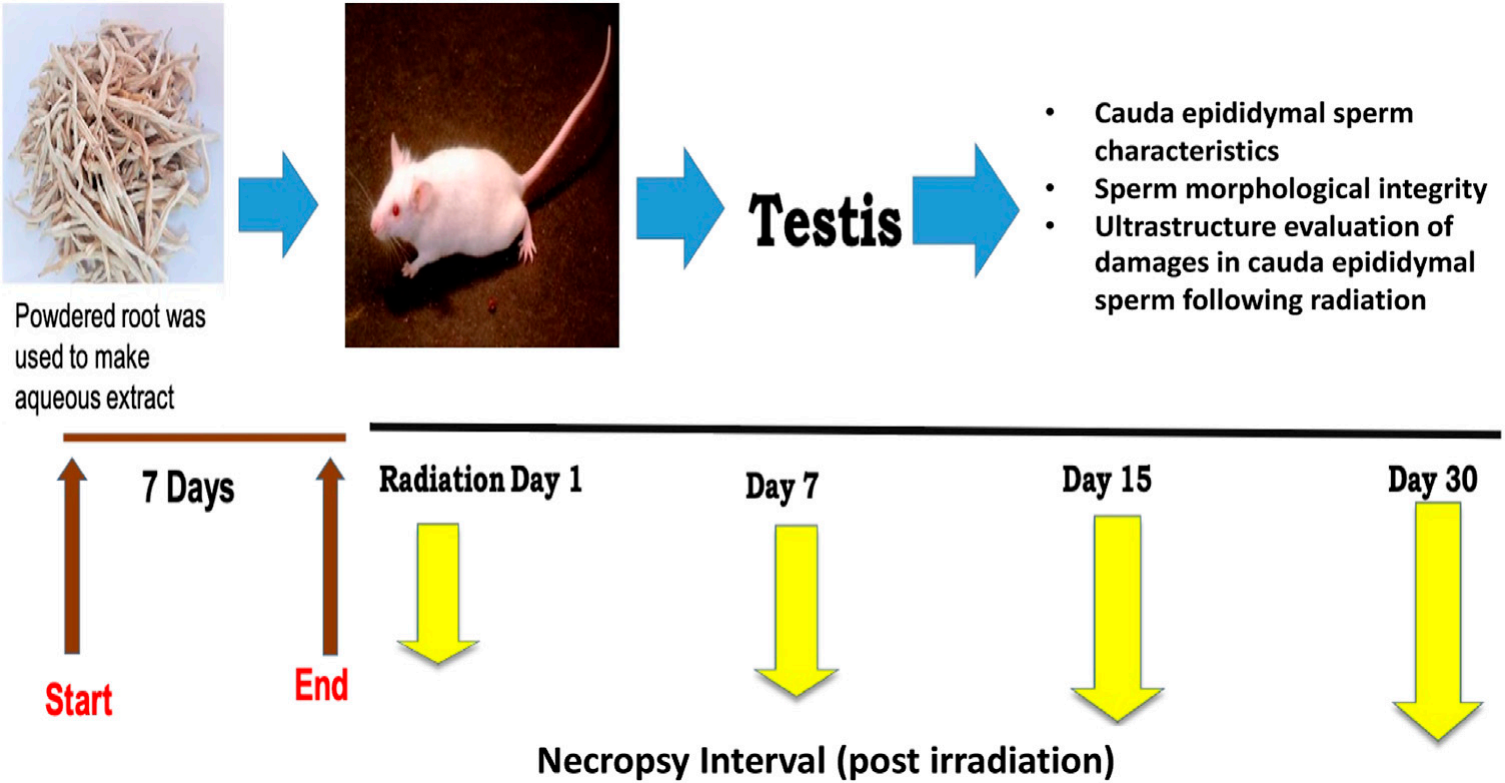
Study design and experimental groups along with measurement parameters.

Based on our preliminary studies (Singh et al., 2018; Vyas et al., 2022), the dose of CRE was set to 50 mg/kg body weight for optimal efficacy. The extract was dissolved in distilled water before administration through oral gavage. The mice were randomly divided into four groups with five animals each, and each group was given 7 days of pre-treatment prior to irradiation (Figure 1). On day 7 of pre-treatment, mice were exposed to 6 Gy whole-body gamma radiation. The four groups were labeled as follows: Group I: control, Group II: 6 Gy gamma radiation-treated positive control, Group III: CRE-treated control, and Group IV: pre-treatment with CRE followed by 6 Gy gamma irradiation. Control animals were given distilled water of equal volume. Animals were observed for 30 days after 7 days of pre-treatment with the plant root extract (CRE). At the end of the investigation schedule, animals were sacrificed by cervical dislocation.

### 2.4 Irradiation by cobalt teletherapy

Animals were irradiated using an ACT-C9 Cobalt Teletherapy Unit, available at the Radiotherapy Department, SMS Medical College and Hospital, Jaipur, India. Whole-body gamma radiation was applied by restraining animals in a well-ventilated Perspex box. The dosimetry was calculated.

### 2.5 Radical scavenging properties

DPPH (1, 1-diphenyl 1-2-picrylhydrazyl) scavenging ability was estimated according to Shimada et al. (1992). Briefly, 2.5 ml of DPPH solution (80 μg/ml) was added to an equal volume of sample solution. The sample solution was prepared with the following concentrations: 10, 50, 100, 200, and 400 μg/ml. Absorbance at 517 nm was measured, followed by incubation of the mixture for 30 min at room temperature. Absorbance was recorded at an interval of 1 h for a maximum of 5 h. The percentage DPPH radical scavenging effect was calculated as follows:

$$DPPH\;scavenging\;effect\;\left(\%\right)=100\left(A_{0}-A_{1}/A_{0}\right).$$

where A_0_ represents the absorbance of the control reaction and A_1_ represents the absorbance in the presence of the standard sample.

IC_50_ was calculated based on the graph curve and percentage inhibition at specific concentrations of CRE.

Nitric oxide (NO) scavenging activity can be estimated by employing the “Griess–Ilosvay” reaction (Garrat, 1964). At the physiological pH (7.2), sodium nitroprusside in an aqueous solution generates NO. Under aerobic conditions, NO reacts with oxygen to produce stable products (nitrate and nitrite), which can be determined using the Griess reagent. Scavengers of NO compete with oxygen, leading to reduced production of nitrite ions, whose absorbance at 540 nm was measured using a UV spectrophotometer. Similar to DPPH, the concentrations of 10, 50, 100, 200, and 400 μg/ml of CRE were estimated for NO-scavenging ability, and absorbance at 540 nm was recorded at 1 h intervals for a maximum of 5 h. The calculation for the scavenging effect and IC_50_ of NO was the same as the DPPH calculations.

### 2.6 Sperm quality parameters

To examine sperm quality parameters, the cauda epididymis was dissected, and tissue samples were cut off and placed in 1 ml saline solution. A drop of the sample was poured over the slide, and sperm count, motility, viability, and abnormality were recorded. For calculations, methods were carried out according to the WHO manual (2010). Sperm samples were examined further by Papanicolaou staining (WHO, 2010).

### 2.7 Ultrastructure of cauda epididymal sperm

Cauda epididymal sperm samples were prepared for scanning electron microscopy (SEM) according to Aladakatti and Ahamed (1999). Sperm samples were fixed in 2% glutaraldehyde and centrifuged at 600 x g for 5 min. Samples were washed carefully with 0.1 M sodium cacodylate buffer (pH 7) and finally washed with distilled water. Furthermore, a film of the solution was placed onto a coverslip. Later, this sample was air-dried and coated with gold before observation under a scanning electron microscope (Zeiss EVO 18, Jena, Germany).

### 2.8 Statistical analysis

Dose–response curves for both DPPH and NO were plotted in Microsoft Excel (Microsoft, US). The linearity relationship was measured by a straight line between variables. Numeric values were expressed in mean ± SEM. Parametric analysis was carried out using Student’s *t*-test (Microsoft, US).

## 3 Results

### 3.1 DPPH- and NO-scavenging effect

**FIGURE 2:**
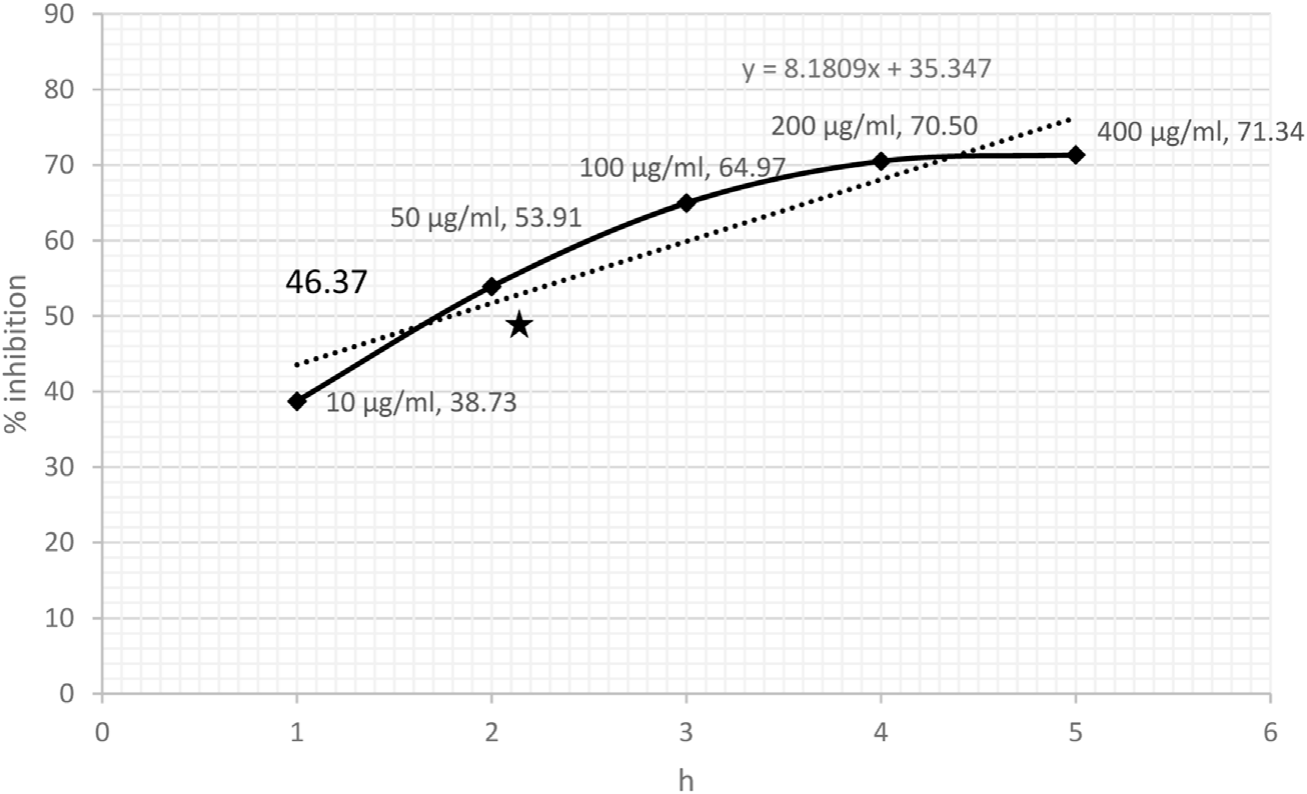
DPPH-scavenging effect against various concentrations of *C. borivilianum* root extract (CRE). IC_50_ was estimated based on the graph of respective doses.

**FIGURE 3:**
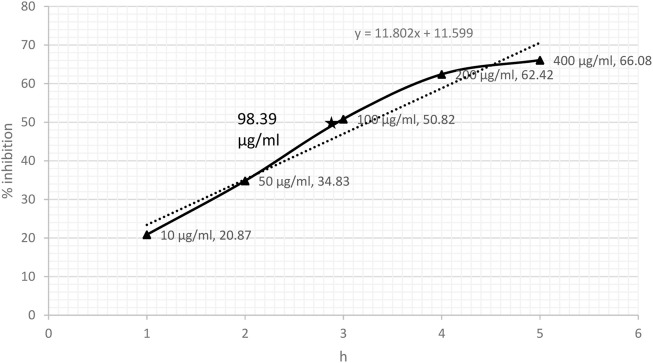
Nitric oxide radical scavenging assay against various concentrations of *C. borivilianum* root extract (CRE). IC_50_ was estimated based on the graph of respective doses.

DPPH-scavenging ability indicated a steady increase based on the concentration of CRE. The results showed 38.73% inhibition at a concentration of 10 μg/ml and became stationary at 200 μg/ml, reaching an optimum of 70.50%. The stationary effect remained constant despite increasing the dose up to 400 μg/ml (Figure 2). The IC_50_ of CRE for the DPPH-scavenging potential was 46.37 μg/ml. The NO radical scavenging assay indicated a similar pattern; however, the stationary effect was not reached until 400 μg/ml concentration. Maximum inhibition of 66% was noted at 400 μg/ml although inhibitory activity was only minutely elevated compared to that at 200 μg/ml concentration, which was 62.42% (Figure 3). Finally, IC_50_ of CRE for NO was 98.39 μg/ml, which was almost two-fold higher than DPPH-scavenging activity.

### 3.2 Cauda epididymal sperm characteristics

Animals exposed to radiation indicated a severe decline in sperm count, motility, and viability (*p* < 0.01). However, animals pre-treated with CRE (Group IV) showed a relatively lower decline than those in Group III. Animals treated with CRE only (Group II) showed no significant alteration in sperm count, motility, and viability. Abnormality in sperm increased substantially in groups III (51.57 ± 4.61%) and IV (47.29 ± 4.21%) (*p* < 0.001). Animals pre-treated with CRE contained relatively lower abnormalities than those in Group III (Table 1).

**TABLE 1:** Cauda epididymal sperm characteristics in animals with and without pre-treatment with *C. borivilianum* root extract (CRE). The asterisks are levels of significance against Group I, where *p < 0.05, **p < 0.01, and ***p < 0.001.

Group	Sperm count (mil/ml)	Sperm motility (%)	Sperm viability (%)	Sperm morphological abnormality (%)
Group I	46.32 ± 5.83	66.72 ± 4.89	84.75 ± 6.27	25.59 ± 2.73
Group II	48.79 ± 6.29	66.55 ± 5.09	83.39 ± 6.88	24.49 ± 3.11
Group III	13.76 ± 1.8***	32.41 ± 4.25***	48.39 ± 3.89***	51.57 ± 4.61***
Group IV	31.76 ± 2.89**	40.61 ± 3.66**	62.69 ± 4.03*	47.29 ± 4.21***

### 3.3 Sperm morphological integrity

**FIGURE 4:**
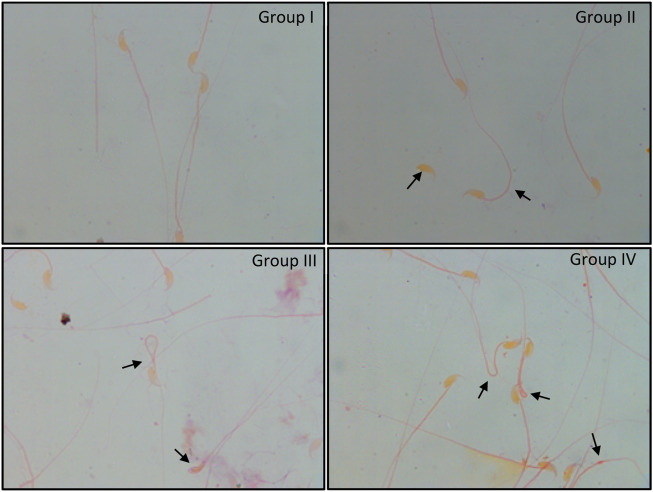
Papanicolaou staining of cauda epididymal sperm exposed to 6 Gy gamma irradiation pre-treated with or without *C. borivilianum* root extract (CRE). Typical coiled and bent tail, head–tail separation, broken tail, hairpin tail, cytoplasmic droplet, and amorphous head are shown. Magnification: ×100.

Normal morphology of cauda epididymal sperm was apparent in groups I and II. These sperm showed typical characteristics with minimal abnormalities, whereas irradiated mice (Group III) showed significantly higher abnormalities that included bent neck, head and tail separation, flattened head, coiled tail, and cytoplasmic droplets (Figure 4). In Group IV, similar abnormalities were noted in sperm. However, the number of abnormalities was relatively lower than that in Group III.

### 3.4 Ultrastructure evaluation of damage in cauda epididymal sperm following irradiation

**FIGURE 5:**
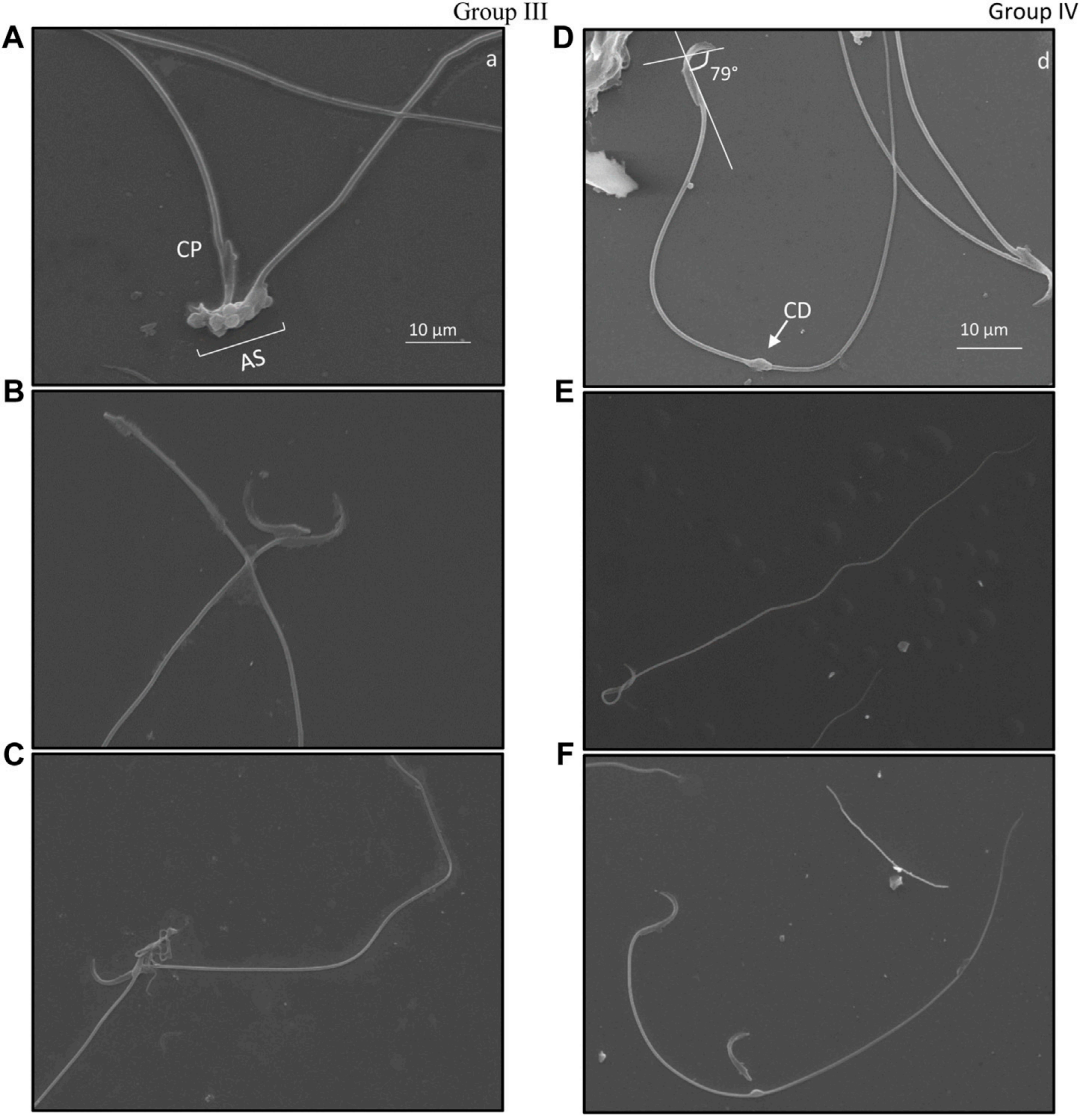
Ultrastructure of cauda epididymal sperm indicating types of damage exclusively present in mice exposed to 6 Gy gamma irradiation. Damage was primarily found in the acrosomal system (AS) where angular deviation of the perforatorium (P) was higher than that of the outer periphery but lower than that of the ventral periphery. Ventral spur (VS) led to the angular bend in the apical (A) acrosomal system. The connecting piece (CP) was dislocated due to head and tail separation. Cytoplasmic droplets (CD) were common in the plasma membrane; these were found in both head and tail sections. Group III, **(A**–**C)**; Group IV, **(D**–**F)**.

Specific structural changes in cauda epididymal sperm indicated distortion of the hook-shaped head. A comparative observation between ultrastructural morphology of cauda epididymal sperm in groups III and IV was studied. Angular evaluation of the head cap system and ventral spur indicated high dissimilarities in the curvature of the hook (Figures 5B, D, F). Noticeably, angular changes in groups III and IV slightly varied. It was observed that Group III (Figure 5B) sperm had a higher angle between the head cap system and ventral spur than Group IV sperm (Figures 5D, F). Twisting of the neck from the middle piece and bending in the tail was eased in Group IV (Figure 5E) compared to Group III (Figure 5C). Cytoplasmic droplets of sizes 1 µm were apparent in the sperm tail of most irradiated mice. Surprisingly, most droplets were present at the mid-area of the tail. Nonetheless, cytoplasmic remnants were also excessively present in the acrosomal system and neck area, indicative of peroxidative damage (Figure 5A). Head–tail separation was predominantly found due to the dislocation of the connecting piece. The presence of amorphous and tapered sperm in the cauda epididymis is indicative of toxic encounters and/or interference in spermatogenesis (Figure 5C).

## 4 Discussion

Giribabu et al. (2014) investigated the effect of CRE on diabetic rats for the potential prevention of sperm impairment due to oxidative stress. Authors reported that administration of 250 and 500 mg/kg/day of CRE (aqueous) could effectively lower sperm LPO, hydrogen peroxide, and NO levels while increasing the levels of SOD, CAT, and GPx. They concluded that CRE can effectively prevent oxidative stress, avoiding apoptosis and impairment of sperm characteristics. A previous study by our group also revealed an elevation in activities of GST, GR, and GPx in testicular tissues of irradiated animals pre-treated with CRE (Vyas et al., 2022). CRE maintains homeostasis between emerging radicals and counteracting antioxidants, thus establishing better tolerance and ameliorating damage caused by radiation. It is well established that CRE has antioxidant properties and plays an important role in protecting germ cells against high oxidative stress.

Under excessive stress of oxidative radicals, sperm face a high risk of structural and functional damage (Sabeti et al., 2016; Dutta et al., 2019). Previous studies reported that oxidative stress causes damage to the axoneme and midpiece (Agarwal et al., 2014; Kurkowska et al., 2020). Alahmar (2019) provided evidence that strongly relates oxidative stress with male infertility. The present study evaluated the impact of ionizing gamma radiation on sperm morphological and functional characteristics. Ionizing radiation can replicate and amplify oxidative damage to sperm by many folds as compared to normal oxidative damage. This damage is mostly associated with surface proteins and midpiece of sperm. A previous study explained that ROS can attack lipids, proteins, and cellular communication of sperm during spermiogenesis and epididymal capacitation (Agarwal et al., 2014). Persistent radicals can cause retention of cytoplasmic droplets around the midpiece and may involve in the arrest of spermatogenesis (Rengan et al., 2012). A previous study by our group indicated that CRE could resist testicular damage significantly against radiation insult (Vyas et al., 2020). Cauda epididymis is the distal portion of the scrotum and stores mature sperm awaiting ejaculation. There are sources of evidence showing ionizing radiation-induced functional and morphological alterations in mature sperm (Kesari and Behari, 2012; Gorpinchenko et al., 2014; Kumar et al., 2014). In the present study, we evaluated differentiation in cauda epididymal sperm impairments following radiation treatment and the subsequent ameliorating effect of CRE.

The half-maximal inhibitory concentration of ethanolic CRE for DPPH and NO was remarkable and observed at <50 and <100 μg/ml, respectively. Visavadiya et al. (2010) reported that based on superoxide-, hydroxyl-, DPPH-, and NO-scavenging ability, CRE exhibits extraordinary antioxidant potential. Another study by Govindarajan et al. (2005) reported that ethanolic tuber extract of *C. borivilianum* could efficiently scavenge nearly 85% DPPH at 100 μg/ml concentration. In the present study, CRE could scavenge almost 65% DPPH, indicating a robust antioxidant potential.

The strong antioxidant potential of CRE was evident in cauda epididymal sperm characteristics of animals pre-treated with CRE and subsequent irradiation. Spermatogenesis is a continuous process; thus, it continues to produce sperm regardless of severe interventions such as irradiation. Newly formed sperm are pushed through the rat testis to the caput, corpus, and ultimately to cauda epididymis. In the present study, following 7 days of pre-treatment with CRE, animals were irradiated only on day 7 of the experiment. Later, the animals were observed for distinct differences in sperm quality parameters following a recovery period of 30 days. Certainly, abnormalities reaching the cauda epididymis during and following the recovery period would have distinct damage. Thereafter, the results showed a substantial increase in sperm count, percentage motility, and viability compared to the irradiated group not pre-treated with CRE. Although these parameters were still significantly lower than those of the sham control group, 7-day pre-treatment with the extract provided better resistance against oxidative damage. These results were in accordance with a study carried out by Giribabu et al. (2014) who reported that sperm count and viability in diabetic rats improved significantly following the administration of CRE (aqueous). Likewise, Das et al. (2016) revealed that administration of 125 and 250 mg/kg body weight of CRE could increase both sperm count and motility in normal rats. According to Das et al., *C. borivilianum* elevates spermatogenic activity. It is important to note that the increase in sperm count, motility, and viability could be a result of elevated spermatogenesis rather than higher survival of *de novo* sperm against oxidative damage or spermatogenic interference. The present observations could also be a result of a combination of both the survival of mature sperm and acceleration of spermatogenic events. Interestingly, Das et al. also noted an increase in sperm abnormalities in those normal rats administered with CRE; however, the increase in abnormality was merely 10% higher than that in non-treated animals. The present study noted a slight improvement in the percentage of sperm abnormality (4% decline); however, it was not as substantial as other parameters, such as sperm count, motility, and viability. This reflects that CRE causes a significant increase in testicular functions mainly by accelerating spermatogenesis. Likewise, with spermatogenesis, the number of abnormalities in mature sperm subsequently rises.

Anyway, oxidative stress can impose multiple structural damage in mature sperm such as head–tail separation, damage to the acrosome, loss of segmented columns, degeneration of the microbial sheath, loss of the plasma membrane, and a numerical aberration in the centriole of the neck (Lohiya et al., 1998). Interestingly, a prospective male contraceptive named “RISUG” is entirely based on this concept (Lohiya et al., 2014). The present study noted an increased number of bent tails, separated heads, tail coiling, amorphous heads, and tails without a head in irradiated animals without any prior treatment with CRE. Similar morphology was apparent in irradiated mice with prior CRE treatment; nonetheless, the degree and number of such sperm were relatively lower. Recorded abnormalities in cauda epididymal sperm reflected both spermatogenic error (amorphous sperm) and the effect of excessive oxidative radicals on mature sperm (coiled tail). Observing the ultrastructure of sperm in both groups (with or without CRE treatment) would reveal the mode of action and/or weightage of response to spermatogenic events and sperm maturation.

Cytoplasmic droplets all around the acrosomal system and neck region explained excessive LPO induced by ionizing radiation. The results of this study suggested that acrosomal areas were extremely affected by ionizing insults. It is to be noted that the plasma membrane is targeted by ROS due to the concentration of lipids, especially polyunsaturated fatty acids (Ayala et al., 2014). de la Haba et al. (2013) reported that excessive oxidative stress causes alterations in membrane fluidity. Volatility in membrane fluidity causes the formation of blisters around the acrosomal area. Flesch et al. (2001) reported an association between cytoplasmic droplets and LPO of the plasma membrane. Authors suggested that unregulated membrane fluidity causes the formation of cytoplasmic droplets. However, it was interesting to note that not all sperm simultaneously indicated the formation of similar degrees of cytoplasmic droplets; similarly, its presence was also infrequent in tails. During sperm capacitation, the acrosomal system goes through significant changes in surface proteins and sperm lipids. The process involves loss of cholesterol, destabilization of the membrane, and exposure of phospholipids. These changes are not similar in all sperm that pass from the caput epididymis to cauda epididymis, thus providing a unique identity and fertilizing ability. This could be a potential reason for some sperm being less affected by the incumbent oxidative stress. However, these radical stresses may affect morphological attributes of the head.

The perinuclear theca (PT) surrounds the nucleus of the sperm, which is divided into two distinct regions: the sub-acrosomal layer and the post-acrosomal sheath (Protopapas et al., 2019). This area plays an important role during capacitation and epididymal maturation. According to Hamilton et al. (2019), PT is anchored with glutathione-S-transferase omega 2, which plays a significant role during the epididymal maturation of sperm. Under excessive oxidative stress, PT is likely to deform as it is made up of condensed cytosolic proteins that are structurally rigid (Manandhar et al., 2009). Such damage may alter the shape of the apical hook and its curvature, which is associated with swimming efficiency (Varea-Sánchez et al., 2016). In the present study, the curvature of the apical hook was measured in irradiated mice, which indicates a significant variation between animals administered with CRE and those without treatment. It appears that the angle between the head cap system (HCS) and ventral spur (VS) slightly declined in animals pre-treated with CRE in comparison to animals irradiated without prior treatment. Furthermore, ionizing radiation seemingly reduced the rigidity of PT, resulting in an increase in the angle between HCS and VS, whereas administration of CRE resisted damage to PT. This confirms that not only during spermatogenic events but also during epididymal maturation and storage, CRE protects the structural attributes of sperm. However, administration of CRE prior to radiation does not eliminate morphological abnormalities such as the presence of a coiled tail, bent neck and tail, and cytoplasmic droplets in the tail, which were still present. Nonetheless, damage to the acrosomal system could greatly reduce sperm from fertilizing an egg; thus, minimizing such aberration would enhance the fertility rate.

The present study concluded that CRE (ethanolic) played a protective role in maintaining sperm quality parameters. Sperm epididymal capacitation is important for hyperactivity of sperm and subsequent fertilization. The results of the present study indicate that the administration of CRE could defend sperm from oxidative stress induced by ionizing gamma radiation during the process of epididymal maturation. CRE showed remarkable antioxidant potential, thus suggesting CRE’s role in improving tolerance against oxidative damage both during spermatogenesis and sperm maturation. A more detailed investigation of its role in epididymal capacitation would provide further knowledge in the modulation of the functional capability of mature sperm.

## Data Availability

The raw data supporting the conclusion of this article will be made available by the authors on request.
